# Volvulus gastrique aigu compliquant une hernie diaphragmatique congénitale chez un nourrisson de 3 mois: à propos d’un cas

**DOI:** 10.11604/pamj.2022.42.238.34517

**Published:** 2022-07-27

**Authors:** Takwa Mili, Yosra Ben Ahmed, Intissar Chibani, Tarek Boukesra, Faouzi Nouira, Awatef Charieg, Said Jlidi

**Affiliations:** 1 Service de Chirurgie Pédiatrique « B », Hôpital d´Enfants de Tunis, Faculté de Médecine de Tunis, Université Tunis El Manar, Tunis, Tunisie

**Keywords:** Volvulus, estomac, diaphragme, enfant, cas clinique, Volvulus, stomach, diaphragm, child, case report

## Abstract

Le volvulus gastrique est un mode de révélation rare de la hernie diaphragmatique congénitale. Il s´agit d´une pathologie rare et de diagnostic difficile en pédiatrie. Nous rapportons le cas d´un nourrisson de trois mois ayant présenté une dyspnée aiguë d´aggravation rapide. La radiographie thoracique a montré une large clarté thoracique avec une poche à air gastrique ascensionnée. La tomodensitométrie thoraco-abdominale a objectivé un volvulus gastrique sur une hernie diaphragmatique congénitale gauche. Le traitement chirurgical a consisté en une dévolvulation gastrique puis réduction complète des viscères herniés et fermeture du défect diaphragmatique. L´évolution était favorable. La hernie diaphragmatique congénitale compliquée par un volvulus gastrique doit être considérée comme une urgence diagnostique et thérapeutique et ce du fait du risque de nécrose gastrique menaçant le pronostic vital.

## Abstract (en)

Gastric volvulus is a rare mode of revelation of congenital diaphragmatic hernia. This is an uncommon condition that may be difficult to diagnose in the paediatric population. We here report the case of a three-month-old infant presenting with a rapid worsening of acute dyspnoea. Chest X-ray showed image clarity and ascending gastric air sac. Thoraco-abdominal CT scan showed gastric volvulus complicating left congenital diaphragmatic hernia. Surgical treatment consisted of gastric devolvulation followed by complete reduction of the herniated viscera and closure of the diaphragmatic defect. Patient´s outcome was favourable. Congenital diaphragmatic hernia complicated by gastric volvulus should be considered as a diagnostic and therapeutic emergency due to the risk of life-threatening gastric necrosis.

## Introduction

Le volvulus gastrique est défini par une rotation anormale supérieure à 180° de tout ou une partie de l´estomac par rapport à l´un de ses axes [1]. Ce diagnostic est rare en pédiatrie et de diagnostic difficile. Cette affection est le plus souvent favorisée par un défaut des ligaments de fixation gastrique. L´anomalie ligamentaire peut être soit primitive soit secondaire à d´autres malformations congénitales dont la plus fréquente est la hernie des coupoles diaphragmatiques [2]. Le volvulus gastrique représente une urgence diagnostique et thérapeutique pouvant aboutir dans les formes aiguës à un étranglement avec un risque d´ischémie et de nécrose gastrique. Nous rapportons le cas d´un nourrisson de 3 mois ayant présenté une détresse respiratoire révélant une hernie diaphragmatique congénitale (HDC) compliquée d´un volvulus gastrique intrathoracique. L´intérêt de ce cas clinique est notamment de décrire les symptômes permettant d´évoquer ce diagnostic, de souligner l´apport des techniques d´imagerie dans la démarche diagnostique et d´étayer la prise en charge chirurgicale.

## Patient et observation

**Présentation du patient**: un nourrisson de sexe féminin, issue d´une grossesse bien suivie, est née à terme par césarienne avec un poids de 3700 g. Elle avait une bonne croissance pondérale et ne présentait auparavant pas de symptomatologie respiratoire ni digestive. A l´âge de 3 mois, elle a été amenée aux urgences de pédiatrie pour dyspnée aiguë. Les parents rapportaient une symptomatologie faite de geignement, pâleur et refus de tétée apparue depuis 48 heures.

**Figure 1:**
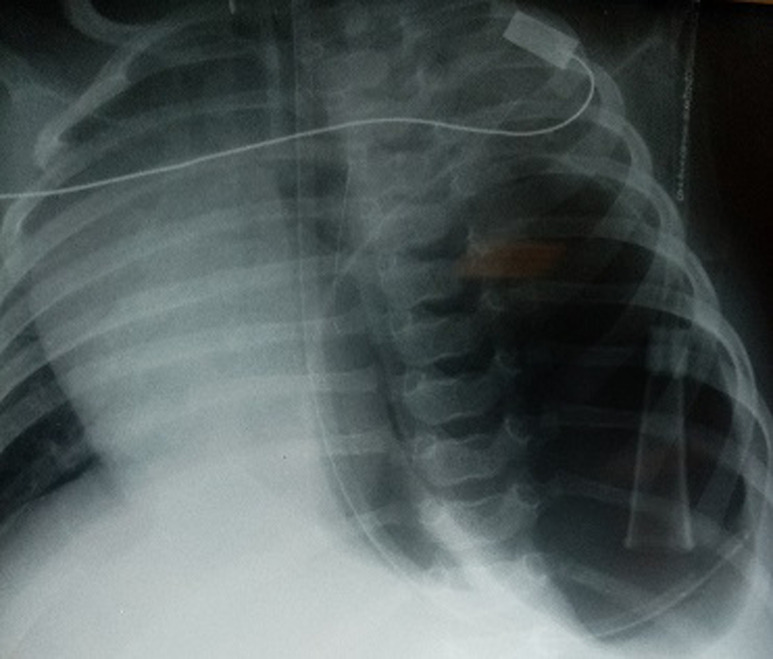
radiographie thoracique de face montrant une large clarté de l´hémi champ pulmonaire gauche avec enroulement de la sonde nasogastrique en intra-thoracique

**Résultats cliniques**: l´enfant était apyrétique et pesait 6100 g. Elle était polypnéique à 67 cycles/minute avec des signes de lutte respiratoire marqués. L´auscultation cardio-pulmonaire trouvait une diminution des murmures vésiculaires dans l´hémi thorax gauche avec déplacement des bruits du cœur à droite. L´abdomen était plat. L´enfant a été hospitalisée et mise sous lunettes d´oxygène à haut débit. La radiographie thoracique de face a montré une large clarté de l´hémi champ pulmonaire gauche avec enroulement de la sonde nasogastrique en intrathoracique. Il n´y avait pas d´image de poche à air gastrique en sous-diaphragmatique et le médiastin était dévié vers la droite (Figure 1).

**Figure 2:**
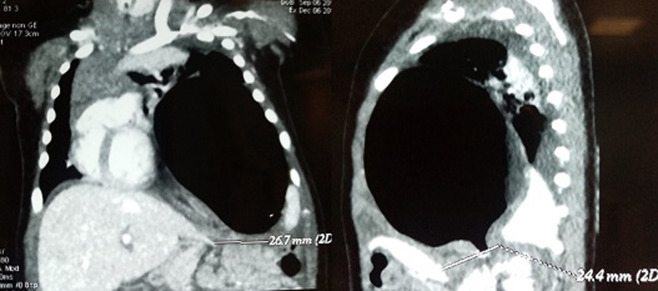
tomodensitométrie thoracique - interruption de la continuité de la coupole diaphragmatique gauche avec estomac intrathoracique volvulé et très distendu et bascule du médiastin vers la droite

**Chronologie**: devant l´aggravation de l´état respiratoire avec apparition d´une cyanose et une hypotonie généralisée, elle a été intubée et transférée en milieu de réanimation. Démarche diagnostique: Une tomodensitométrie thoracique a montré l´interruption de la continuité de la coupole diaphragmatique gauche. L´estomac était complètement ascensionné en intrathoracique. Il était volvulé autour de son axe et très distendu responsable d´une bascule médiastinale à droite (Figure 2).

**Figure 3:**
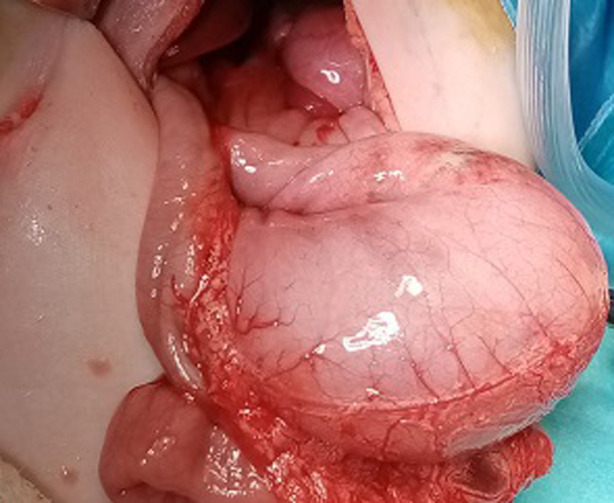
aspect per opératoire montrant un volvulus gastrique organo-axial avec des lésions nécrotiques pré-perforatives au niveau du fundus

**Figure 4:**
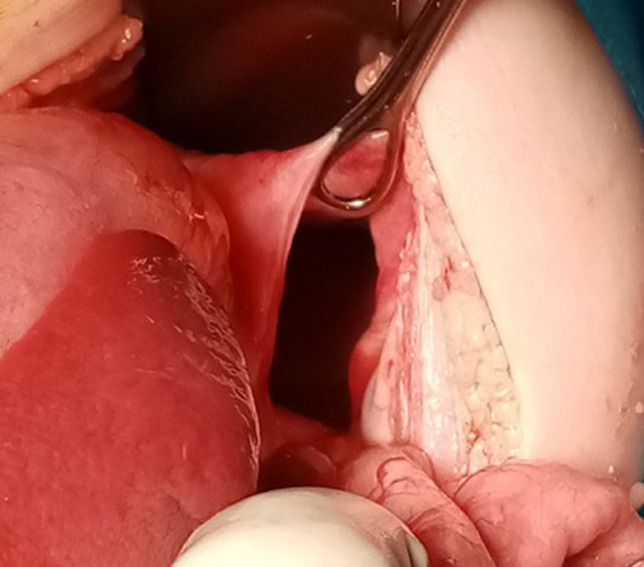
aspect per opératoire - défect diaphragmatique congénital postéro-latéral gauche, de type Bochdalek

**Intervention thérapeutique**: l´exploration chirurgicale par voie médiane sus-ombilicale avait confirmé le diagnostic de volvulus gastrique organo-axial secondaire à une hernie diaphragmatique postéro-latérale gauche, de type Bochdalek. L´estomac était en totalité intrathoracique et présentait une bonne vitalité après réduction du volvulus. Des lésions nécrotiques pré-perforatives ont été constatées au niveau du fundus (Figure 3 et Figure 4). La rate a été également retrouvée ascensionnée en intrathoracique. Le poumon gauche était hypoplasique. Le traitement a consisté en une dévolvulation gastrique puis réduction complète des viscères herniés et fermeture du défect diaphragmatique. L´absence de malrotation intestinale a été vérifiée avant la fermeture de la paroi.

**Suivi et résultats**: les suites opératoires ont été simples. L´enfant a été alimentée à J 7 post-opératoire et mise sortante à domicile à dix jours post-opératoires. Les contrôles ultérieurs ont montré une évolution favorable.

**Consentement du patient**: le consentement éclairé du parent a été obtenu pour utiliser les données iconographiques du patient.

## Discussion

Le volvulus gastrique est rare en pédiatrie. Il peut être un des modes de révélation des HDC de découverte post-natale [1]. Il existe deux formes principales de volvulus gastriques en fonction de l´axe de rotation: le volvulus organo-axial et le volvulus mésentérico-axial. Dans la première, dite organo-axiale, la rotation s´effectue autour de l´axe cardio-pylorique réalisant un vrai volvulus. Il s´agit de la forme la plus fréquente, représentant environ 60 % des cas [3]. Il survient souvent sur une hernie hiatale ou diaphragmatique et se complique fréquemment de strangulation. Dans la deuxième, plus rare et appelée mésentérico-axiale, la rotation s´effectue suivant l´axe longitudinal du petit épiploon. L´estomac est normalement fixé par ses quatre ligaments: le gastro-phrénique, le gastro-splénique, le gastro-hépatique et le gastro-colique. L´absence ou l´hyperlaxité de ces ligaments peut être à l´origine d´une mobilité anormale de l´estomac prédisposant au volvulus. Ce déficit ligamentaire peut être dans 30 % des cas congénital définissant le volvulus gastrique primaire. Ce volvulus peut également être secondaire à d´autres affections, dont la plus fréquente est la hernie congénitale des coupoles diaphragmatiques, comme c´est le cas dans notre observation. L´augmentation de l´espace sous diaphragmatique associée au défect, permet la rotation de l´estomac lors de son ascension en situation intrathoracique. Il s´y associe le plus souvent une anomalie des ligaments de fixation gastrique car deux des quatre ligaments fixant l´estomac (le ligament gastro- phrénique et le ligament gastro-splénique) sont fixés sur la coupole diaphragmatique gauche. Le volvulus gastrique est secondaire plus de deux fois sur trois en pédiatrie et l´association volvulus gastrique secondaire et HDC atteint 42% dans certaines séries [4].

Chez l´adulte, le diagnostic de volvulus gastrique est souvent porté devant la triade de Borchardt: distension gastrique et douleur épigastrique, vomissements incoercibles, impossibilité de mettre en place une sonde gastrique. Cette triade a rarement été décrite chez l´enfant [4], chez lequel le diagnostic est le plus souvent porté devant des manifestations aiguës liées à une occlusion digestive haute (vomissements alimentaires post- prandiaux précoces). Sont parfois associés des symptômes respiratoires en fonction de l´importance du défect diaphragmatique ou une instabilité hémodynamique si l´enfant est vu tardivement. Dans notre observation, le tableau clinique était dominé par les signes respiratoires en rapport avec le défect diaphragmatique, l´hypoplasie pulmonaire et la compression thoracique par l´estomac distendu. Les examens radiologiques ont une importance capitale dans le diagnostic du volvulus gastrique. Le cliché thoraco-abdominal demandé surtout dans un contexte aigu peut montrer une anomalie de position de l´estomac avec un trajet aberrant de la sonde nasogastrique ou des niveaux hydro-aériques. Cet aspect radiologique associé à un tableau d´obstruction digestive haute chez un nourrisson doit faire suspecter le diagnostic et indiquer une prise charge chirurgicale immédiate. Le transit œsogastroduodénal est l´examen essentiel pour le diagnostic des volvulus gastriques. Il est réalisé en l´absence de choc cardiorespiratoire, de péritonite ou de médiastinite où l´indication chirurgicale ne se discute pas. Cet examen permet de confirmer le diagnostic du volvulus gastrique, sa position, sa forme anatomique mésentérico-axiale ou organo-axiale et l´évacuation antropylorique du produit de contraste [5]. Cet examen n´a pas été réalisé pour notre patiente vue la gravité de la détresse respiratoire. Du fait de la rareté de cette maladie, l´apport de la tomodensitométrie n´est actuellement pas bien établi bien que cet examen soit d´un grand intérêt dans le diagnostic positif permettant de préciser le type de volvulus, sa cause et surtout les conséquences cardiopulmonaires de la migration intrathoracique. Les reconstructions sagittales permettent de préciser le sens de la bascule gastrique, les points de torsion et les facteurs anatomiques favorisants.

En cas de retard diagnostique dans ces formes aiguës, le volvulus gastrique peut se compliquer d´une dilatation extrême de l´estomac et de sa nécrose ou rupture, la mortalité pouvant alors atteindre 65% [6]. La chirurgie est le traitement de choix du volvulus gastrique. Elle a pour but la dévolvulation et la réintégration de l´estomac, le traitement de l´étiologie et la prévention des récidives. En cas de volvulus gastrique aigu secondaire à une hernie de la coupole diaphragmatique comme c´est le cas dans notre observation, le traitement chirurgical consiste en la dérotation de l´estomac et la fermeture du diaphragme. La gastropexie a pour but de fixer l´estomac aux structures de voisinage afin de contrecarrer la flaccidité des attaches gastriques qui représentent l´élément anatomique fondamental dans la genèse du volvulus et ainsi d´éviter les récidives. Plusieurs types de fixation ont été décrits notamment la gastropexie antérieure, qui est la plus utilisée, amarrant la face antérieure de l´estomac à la paroi abdominale antérieure, la gastropexie postérieure au péritoine pariétal postérieur et la fixation gastrique au diaphragme [7]. Cependant, cette attitude est controversée par certains auteurs qui rapportent de bons résultats à long terme sans gastropexie et ce quelle que soit la voie d´abord conventionnelle ou laparoscopique [8]. Si la prise en charge chirurgicale est précoce, le pronostic des volvulus gastriques révélés par des manifestations aiguës est favorable. Des formes chroniques de volvulus gastriques ont été décrites et diffèrent des formes aiguës par leur présentation clinique. Les symptômes (douleurs abdominales, distension gastrique et vomissements) sont intermittents, s´agissant de formes incomplètes de volvulus qui régressent spontanément mais peuvent récidiver [9]. Certaines équipes chirurgicales optent alors pour l´abstention thérapeutique puisque les symptômes disparaissent le plus souvent spontanément après l´âge d´un an. D´autres équipes préfèrent la gastropexie préventive [10].

## Conclusion

Le volvulus gastrique est un mode de révélation post-natale d´une HDC. Il s´agit d´une entité rare qui doit être considérée comme une urgence diagnostique et thérapeutique et ce du fait du risque de nécrose gastrique menaçant le pronostic vital. Il faut évoquer le diagnostic sur la clinique et surtout sur le cliché thoraco-abdominal. Le transit œsogastroduodénal permet, en absence de détresse vitale, de confirmer le diagnostic. La tomodensitométrie permet de préciser le type de volvulus et sa cause et de dresser le bilan lésionnel thoracique. Le traitement est chirurgical. Les gestes doivent comporter dévolvulation et réintégration de l´estomac hernié et la fermeture du défect diaphragmatique. En l´absence d´études prospectives randomisées, nous ne pouvons affirmer avec certitude que la gastropexie permet de réduire le risque de récidive du volvulus gastrique de l´enfant.
